# Anti-Aβ antibodies bound to neuritic plaques enhance microglia activity and mitigate tau pathology

**DOI:** 10.1186/s40478-020-01069-3

**Published:** 2020-11-23

**Authors:** Vanessa Laversenne, Sameer Nazeeruddin, Emma C. Källstig, Philippe Colin, Christel Voize, Bernard L. Schneider

**Affiliations:** 1 Brain Mind Institute, Ecole Polytechnique Fédérale de Lausanne (EPFL), Station 19, 1015 Lausanne, Switzerland; 2 Bertarelli Foundation Gene Therapy Platform, School of Life Sciences, Ecole Polytechnique Fédérale de Lausanne (EPFL), 1202 Geneva, Switzerland

**Keywords:** Alzheimer’s disease, Tau, Amyloid beta, Microglia, Neuritic plaques, Passive immunization

## Abstract

The brain pathology of Alzheimer’s disease (AD) is characterized by the misfolding and aggregation of both the amyloid beta (Aβ) peptide and hyperphosphorylated forms of the tau protein. Initial Aβ deposition is considered to trigger a sequence of deleterious events contributing to tau pathology, neuroinflammation and ultimately causing the loss of synapses and neurons. To assess the effect of anti-Aβ immunization in this context, we generated a mouse model by overexpressing the human tau protein in the hippocampus of 5xFAD mice. Aβ plaque deposition combined with human tau overexpression leads to an array of pathological manifestations including the formation of tau-positive dystrophic neurites and accumulation of hyperphosphorylated tau at the level of neuritic plaques. Remarkably, the presence of human tau reduces microglial clustering in proximity to the Aβ plaques, which may affect the barrier role of microglia. In this mouse model, continuous administration of anti-Aβ antibodies enhances the clustering of microglial cells even in the presence of tau. Anti-Aβ immunization increases plaque compaction, reduces the spread of tau in the hippocampal formation and prevents the formation of tau-positive dystrophic neurites. However, the treatment does not significantly reduce tau-induced neurodegeneration in the dentate gyrus. These results highlight that anti-Aβ immunization is able to enhance microglial activity around neuritic plaques, mitigating part of the tau-induced pathological manifestations.

**Electronic supplementary material:**

The online version of this article (10.1186/s40478-020-01069-3) contains supplementary material, which is available to authorized users.

## Introduction

In the absence of any disease-modifying treatment, Alzheimer’s disease (AD) leads to the progressive loss of memory and other cognitive functions. Brain pathology is characterized by the accumulation of senile plaques mainly composed of amyloid beta (Aβ) fibrils [19, 48] and neurofibrillary tangles (NFTs) which contain hyperphosphorylated tau protein [7]. The Aβ cascade hypothesis has proposed that Aβ accumulation and deposition are primary pathogenic events leading to major disease hallmarks including tau hyperphosphorylation, microglia activation, as well as the loss of synaptic connections and neuronal cells [22, 62]. Hence, most treatments against AD that have been or are currently tested in clinical trials target the production and deposition of Aβ. Among these, passive immunization using monoclonal antibodies (mAbs) against Aβ has shown some efficacy at clearing senile plaques in the brain of AD patients, and is currently being tested for its ability to slow down cognitive decline [53]. In this context, it is important to further explore the relationship between the Aβ and tau pathologies [46], considering that the level of Aβ deposition does not correlate with cognitive decline [15, 64], and that the mechanistic links between Aβ and tau are not fully elucidated yet.

As overexpressing human wild-type (WT) tau in the mouse species does not necessarily trigger conversion to pathological tau species [14], most studies have addressed this question by overexpressing tau carrying pro-aggregant mutations typically associated with frontotemporal dementia [5, 20, 24, 42, 51, 54, 60]. However, it was recently shown in a mouse model expressing the human tau repeat domain that the presence of neuritic plaques can also promote WT tau conversion into pathological forms [44]. Furthermore, neuritic plaques facilitate the formation of NFTs following brain inoculation with tau seeds derived from human AD brain [23]. These studies highlight the importance of the interaction between Aβ and WT tau at the level of the neuritic plaques in the development of tau pathology.

Here, the 4R0N isoform of human WT tau was locally overexpressed in the hippocampal CA3 region of 5xFAD mice which display rapid deposition of Aβ plaques in the brain [49]. As the overexpressed human tau protein spread throughout the hippocampal formation, this model was found to recapitulate some hallmarks of the disease, including neuronal loss in the CA3 area and dentate gyrus (DG). The combined effects of tau and Aβ induced the formation of tau-positive neuritic plaques, characterized by dystrophic neurites, tau accumulation and hyperphorphorylation, as well as microglial activation. Whereas microglia were typically observed in clusters around Aβ plaques, the overexpression of human tau altered the distribution of microglial cells, reducing their presence near the neuritic plaques.

In this context, we determined the effects of passive anti-Aβ immunization with the recombinant mAb11 antibody continuously delivered following peripheral implantation of encapsulated genetically modified myoblasts. Anti-Aβ antibody binding was found to promote the clustering of CD68-positive microglia around plaques. The treatment increased plaque compaction, reduced the formation of tau-positive dystrophic neurites, and led to an overall decrease in tau immunoreactivity in the hippocampal formation. However, the anti-Aβ immunization had only limited effects on tau-induced neuronal loss.

## Materials and methods

### Animal studies

Animal studies were authorized by a local ethics committee and were performed in accordance with the Swiss Federal Law for animal protection and experimentation (Art.18 LPA, Art. 141 OPAn, Art. 30) under the license numbers VD-3130 and VD-3210.

Experiments were performed using 5xFAD transgenic mice bred on the original hybrid B6SJL background (Jackson Laboratory, B6SJL-Tg (APPSwFlLon, PSEN1*M146L*L286V) 6799Vas/Mmjax, stock No 34840-JAX). These mice carry two transgenes encoding (1) the human amyloid precursor protein (APP) with the Swedish (K670N/M671L), Florida (I716V), and London (V717I) mutations, and (2) the human presenilin 1(PS1) protein with the M146L and L286V mutations [49]. We used only hemizygous 5xFAD mice and their wild-type B6SJL littermate for the present experiments. The experimental groups of mice were age-matched and carefully gender-balanced.

### Anti-Aβ mAb11 antibody

The mAb11 antibody was designed to recognize a conformational epitope of Aβ aggregates. It was derived from the human combinatorial antibody library Fab 1 (MorphoSys; HuCAL-Fab1) [55] and then further optimized through in vitro maturation for plaque binding [4]. Similar to Gantenerumab, the mAb11 antibody has higher affinity for both oligomeric and fibrillar forms than monomeric forms of the Aβ peptide [4]. To allow for secretion of a murinized version of the mAb11 antibody adapted to experiments in mice using the encapsulated cell technology (ECT), two cDNAs encoding the heavy and light chains of a human mouse chimeric version of mAb11 (human variable domains, mouse immunoglobulin 2a, IgG2a, constant domains) were synthetized and subcloned in the pRRLSIN.cPPT.PGK vector. Lentiviral particles were generated to transduce C2C12 myoblasts [34].

### Cell culture

For antibody delivery, we used the antibody-secreting clone#29 derived from the murine C2C12 myoblast cell line and which was obtained as previously described [36]. The original C2C12 cell line was purchased from the American Type Culture Collection (ATCC number CRL-1772). Cells were cultured at 37 °C with 5% CO_2_ in Dulbecco’s modified Eagle Medium supplemented with 10% fetal bovine serum, 100 U/ml penicillin and 100 μg/ml streptomycin.

### Encapsulated cell technology

ECT with a dedicated implant was used for continuous subcutaneous antibody delivery in mice. The devices were assembled by the Cell Encapsulation Technology Platform at Nestlé Institute for Health Science as previously reported [35]. Cells were seeded in the capsules using a polymeric hydrogel biomaterial as extracellular matrix [16, 17, 35]. We used a 10% stoichiometrically balanced mix of two functionalized polyethylene glycol (PEG) precursors: n-PEG-MMP-Lys (W) with the W mutation allowing enzymatic cell degradation and n-PEG-Gln (NQ) ([Lys]/[Gln] = 1). This 10% NQ/W PEG hydrogel was diluted in Tris-buffered saline (TBS 50 mM, pH 7.6) at the desired w/v percentage to reach the wanted stiffness. For the batch used in this study, 1.5% w/v PEG hydrogel corresponded to a stiffness of 300 Pa.

### Implantation

The detailed procedure for cell loading in the implant was already described [35]. The surgery for subcutaneous device implantation was performed under isoflurane anesthesia. Mouse skin was opened between shoulder blades and detached from the muscle using a spatula. Implants were carefully rinsed with sterile PBS before insertion underneath the skin.

### MAb11 quantification with ELISA

The detailed protocol of the ELISA assay used to quantify mAb11 was previously described [34]. Every other week, blood was sampled from the saphenous vein in EDTA-coated tubes (Sarstedt). Plasma was obtained by centrifugation and kept at –80 °C until further analysis. For mAb11 ELISA, the plasma samples were diluted in LowCross Buffer (Candor Bioscience) and incubated for 1 h at RT in 96-well plates coated with 7 μg/ml Aβ peptide. After washing with PBS/0.05% Tween 20, plates were incubated with peroxidase-conjugated goat anti-mouse antibody. After incubation with peroxidase substrate, absorbance was measured at 405 nm using a Versamax plate reader (Molecular Devices). A standard curve was established using purified mAb11 IgG2a and calculated using a non-linear four-parameters fit (SoftMax Pro Software, Molecular Devices).

### AAV production and stereotaxic injection in the hippocampus

The pAAV-PGK-WPRE constructs encoding either GFP or human 4R0N tau protein were previously described [38]. Recombinant AAV8 particles were produced by transient transfection of HEK293-AAV cells (Agilent) and purified using an iodixanol gradient and ion-exchange chromatography according to standard procedures. The concentration of viral genome-containing AAV8 particles (VG) was determined using qPCR with primers specific for the WPRE sequence.

5xFAD and wild-type littermate mice were unilaterally injected with 2.8^E^10 VG in the CA3 hippocampus located in the right hemisphere. We used the following stereotaxic coordinates: antero-posterior − 2.1 mm, lateral − 2.25 mm, ventral − 2.25 mm from the skull surface.

### Necropsy

Animals were overdosed with pentobarbital and a terminal intracardiac puncture was performed to sample blood and obtain plasma for measurement of mAb11 level by ELISA. Implants were carefully dissected from subcutaneous tissue before perfusing the animals with 4% paraformaldehyde and extracting the brains. Collected brains were kept at 4 °C overnight in 4% paraformaldehyde and transferred to a 25% sucrose solution in PBS prior to tissue processing. Implants were incubated overnight at 37 °C in cell culture medium to verify antibody secretion by ELISA. The devices were finally embedded in paraffin for histological processing.

### Immunohistochemistry

After cryoprotection in 25% sucrose solution, 25-µm thick coronal brain sections were cut using a cryostat and kept at 4 °C in PBS plus azide. Prior to immunohistochemistry, sections were mounted on Superfrost plus slides (Thermo Scientific). For all immunohistochemical analyses except for HT7 staining, quantification was performed on one in every eight coronal sections, with an inter-section interval of 200 µm, for a total of 5 to 6 sections per mouse brain. For each animal, all the sections used for quantification were selected within the most anterior one-millimeter thick portion of the hippocampus, according to anatomical landmarks. For quantification of total human tau (HT7 immunohistochemistry), we used in each mouse one in every four coronal serial sections, for a total of 10-12 slices in the same most anterior part of the hippocampus, with an inter-section interval of 100 µm.

The following primary antibodies were used using a standard immunohistochemistry protocol: for Aβ plaques, mouse IgG1 anti-human Aβ antibody clone 6e10 (Sigma 39320, 1:500); for microglia, rabbit anti-mouse Iba1 (Abcam EPR16588, 1:500); for phagolysosomes, rat anti-mouse CD68 (BioRad MCA1957GA, 1:500). As secondary antibodies, we used AlexaFluor 488 goat anti-mouse IgG1 (Jackson ImmunoResearch, 1:500), AF647 donkey anti-rabbit IgG (Jackson ImmunoResearch, 1:250), CY3 Donkey anti-rat IgG (Jackson ImmunoResearch, 1:1000) and CY3 Donkey anti-rabbit IgG (Jackson ImmunoResearch, 1:1000), respectively. Briefly, sections were blocked in 5% normal goat serum or 5% normal donkey serum in 0.1% Triton-X100/PBS for at least 2 h at RT. Primary antibodies were incubated overnight in blocking buffer at 4 °C. Sections were washed with PBS and incubated for 2 h with fluorescent conjugated secondary antibodies. After immunohistochemical staining, sections were labeled with DAPI and the slides mounted with fluoromount-G (SouthernBiotech).

For Aβ staining, we also used the anti-Aβ mouse IgG2b antibody clone 4G8 (Biolegend 800709, 1:1000). In this case, the sections were pre-treated with 70% formic acid for 30 min at RT followed by antigen retrieval at 95 °C for 20 min in 10 mM trisodium citrate buffer. As secondary antibody, we used AlexaFluor 488 goat anti-mouse IgG2b (Jackson ImmunoReseach, 1:500).

For immunohistochemistry to reveal the presence of the human tau protein, we applied antigen retrieval for 20 min at 95 °C in 10 mM trisodium citrate buffer prior to the standard protocol. MC1 antibody against misfolded human tau (kindly provided by Peter Davies, Department of Pathology, Albert Einstein College of Medecine, used 1:500) was incubated overnight and labeled using a CY3 goat anti-mouse IgG1 specific secondary antibody (Jackson ImmunoReseach, 1:500). For detection of total human tau and phospho-tau (Ser202/Thr205 phosphorylation), we used the biotin-conjugated mouse primary antibodies HT7 (Pierce MN1000B, 1:1000) and AT8 (ThermoFisher MN1020B, 1:1000), respectively. The signal was amplified using the AF555 Tyramide SuperBoost Kit (Invitrogen, B40913). For the Gallyas silver staining, we used the modified Gallyas silver impregnation protocol described elsewhere [71].

Aβ plaque immunodecoration with anti-Aβ mAb11 was revealed using a CY3-conjugated goat anti-mouse IgG2a-specific antibody (Jackson Immunoresearch). To avoid non-specific binding, sections were first treated with Ultra V blocking solution (LabVision), washed for 5 min with PBS/0.01% Tween-20 before a second blocking step using Power Block Solution 1x (BioGenex) supplemented with 10% normal sheep serum for 2 h at RT. Sections were then incubated for 1 h with the goat anti-mouse IgG2a-specific antibody (1:100) in 1% Bovine Serum Albumin (BSA).

### Image Processing

After immunohistochemistry, whole brain slices were imaged with a 10x objective using a slide scanner (Olympus, VS120-L100). Images acquired in the virtual slide format (.vsi) were opened and analyzed using the QuPath software [2]. DAPI signal was used to draw regions of interest (ROI) in the ipsilateral and contralateral hippocampal formations using a drawing tablet. The area covered by the fluorescent signal within the ROI was measured using thresholding algorithms in Fiji [61]. The algorithm was chosen to fit the observed signal as accurately as possible. Depending on signal distribution and intensity, either Triangle, Moments or Otsu algorithms were selected for analysis.

Identification of individual Aβ plaques and microglia based on their size was performed on QuPath after signal thresholding using dedicated algorithms. For analysis of signal coverage at the level of Aβ plaques, we defined a ROI including a 5-μm peripheral rim around the Aβ signal using QuPath. The area positive for the signal of interest (Iba1, CD68 or HT7) was obtained by thresholding and the measured surface was divided by the total area of the ROI to determine the percentage of coverage.

High-resolution images of the brain tissue with fluorescent immunostainings were taken either with a Leica DM5500 microscope or with a confocal Zeiss LSM700 microscope.

### Statistical analysis

Graphing and statistical analysis were performed on Prism (GraphPad). Data are represented as mean ± standard error of the mean (SEM) unless specified in the text or legends. Two-tailed unpaired homoscedastic Student’s *t* test was used for comparisons between two groups. For experiments containing more than two groups, one- or two-way ANOVA were applied, and corrected for multiple comparisons using the Sidak post hoc test. The alpha level of significance was set at 0.05 and p values were reported as follows: ns, *p* > 0.05, *p ≤ 0.05, **p ≤ 0.01, ***p ≤ 0.001, ****p ≤ 0.0001.

## Results

### Unilateral injection of AAV-tau in the CA3 hippocampus of 5xFAD mice to generate a model of Aβ/tau pathology

In order to assess the interaction between the Aβ and tau pathologies in vivo, we developed a mouse model based on the unilateral intrahippocampal injection of a serotype 8 adeno-associated viral (AAV8) vector encoding the wild-type 4R0N form of human tau under the control of the mouse Pgk1 promoter (AAV-tau). The vector was injected in the hippocampal CA3 region of 2-months old 5xFAD mice, to overexpress the human tau protein in AD mice rapidly developing the Aβ pathology.

Between 1.5 and 6 months after vector injection, human tau HT7 immunoreactivity was found to spread via axonal projections from the injected ipsilateral hippocampus to the contralateral hippocampus (Additional file 1: Fig. S1a). After 5 months, HT7 immunostaining for human tau revealed the presence of the overexpressed human tau protein throughout the anterior hippocampal formation (Additional file 1: Fig. S1b). Concomitant with the accumulation of human tau, immunohistology revealed in the ipsilateral hippocampus the presence of misfolded tau (detected by the MC1 antibody), tau phosphorylated at Ser202/Thr205 residues (AT8 antibody) as well as tau phosphorylated at Ser396/Ser404 residues (PHF1 antibody) (Additional file 1: Fig. S1c). These findings confirmed the propensity of human tau to form pathological species when overexpressed in the hippocampal formation.

As previously reported, the deposition of Aβ in the 5xFAD mice was coinciding with the formation of neuritic plaques surrounded by microglia positively stained with Iba1 and CD68, a marker of the endosomal/lysosomal compartment (Additional file 2: Fig. S2a). Partial co-localization of CD68 and Aβ was observed by confocal microscopy of neuritic plaques, which may indicate microglial phagocytic activity (Additional file 2: Fig. S2a). Furthermore, these neuritic plaques were surrounded by the abundant presence of the pathological autophagic marker Lysosomal-associated membrane protein 1 (LAMP1) (Additional file 2: Fig. S2b). The formation of dystrophic neurites adjacent to Aβ plaques was revealed by the accumulation of the presynaptic protein synaptophysin (Additional file 2: Fig. S2c). Furthermore, in 5xFAD mice injected with AAV-tau, the HT7 staining showed neuritic accumulations of the human tau protein, mainly visible in the hippocampus contralateral to AAV-tau injection (Additional file 2: Fig. S2d). Silver Gallyas staining used to detect tau paired helical filaments [18] revealed the presence of pathological deposits in the dystrophic dendrites surrounding Aβ plaques (Additional file 2: Fig. S2e). In 5xFAD/AAV-tau mice, Gallyas staining was observed only at the level of neuritic plaques in the hippocampus and not in neuronal cell bodies located near the site of AAV-tau injection.

### Aβ promotes hyperphosphorylation of the WT human tau protein

As previously reported, Aβ pathology can accelerate human mutant tau aggregation and propagation [5, 20, 41, 50]. However, little is known about the effect of Aβ on the spreading of WT forms of human tau in the absence of mutations. To address this question, we compared the induced tau pathology in WT and 5xFAD mice.

**Fig. 1:**
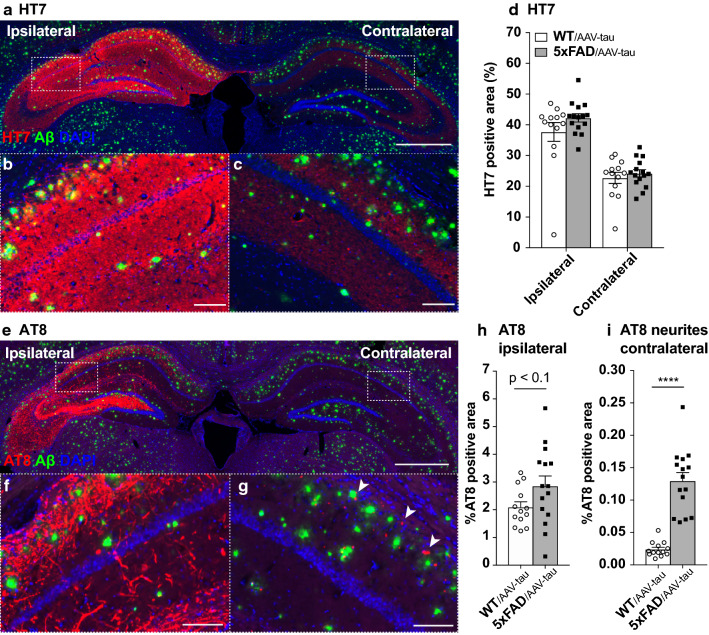
Tau and Aβ pathologies in the 5xFAD/AAV-tau mouse model. **a** Representative image of the distribution of Aβ (4G8, in green) and human tau (HT7, in red) in the hippocampal formation of 5xFAD/AAV-tau mice. **b**, **c** Higher magnification image in the ipsilateral (**b**) and contralateral CA3 (**c**). **d** Percentage of the total hippocampal area covered by HT7 staining in the ipsi- and contralateral hippocampus, in WT/AAV-tau and 5xFAD/AAV-tau mice. **e** Representative image of the distribution of Aβ (4G8, in green) and Ser202/Thr205 phospho-tau immunoreactivity (AT8, in red) in the hippocampus of 5xFAD/AAV-tau mice. **f**, **g** Higher magnification image in the ipsilateral (**f**) and contralateral CA3 (**g**). Note that in the ipsilateral hippocampus, AT8-positive tau is mainly located in the soma and neurites of neuronal cells. In the contralateral hippocampus, AT8-positive tau puncta (arrowheads) are mainly located near amyloid plaques (4G8, in green). **h** Percentage of the hippocampal surface covered by AT8 phospho-tau immunoreactivity in the ipsilateral hippocampus, comparing WT/AAV-tau and 5xFAD/AAV-tau mice. **i** Percentage of the surface covered by AT8 phospho-tau immunoreactivity in the contralateral hippocampus. Note the significant coverage increase in the 5xFAD/AAV-tau mice, which reflects the presence of phospho-tau-positive neuritic puncta. Scale bars: 800 µm (**a**, **e**) and 100 µm (**b**, **c**, **f**, **g**). Statistical analysis: unpaired two-tailed Student’s t-test, ****p < 0.0001; WT/AAV-tau: n = 13 mice, 5xFAD/AAV-tau: n = 15 mice

We quantified the distribution of human tau throughout the hippocampal formation by measuring the hippocampal surface covered by HT7 immunostaining. The human tau protein was highly expressed near the injection site, in the CA3 and DG, and was found in axons projecting towards the CA1 and CA2 areas (Fig. 1a–c). In the ipsilateral hippocampus, the protein was broadly distributed in the somatodendritic and axonal compartments (Fig. 1a, b). In the contralateral hippocampus, human tau was mainly observed within the projecting axons (Fig. 1a, c). On both sides, the fraction of the hippocampal surface covered by the HT7 staining remained similar when comparing WT/AAV-tau and 5xFAD/AAV-tau mice (Fig. 1d; ipsilateral side: 37.7 ± 11.0% for WT/AAV-tau mice and 42.2 ± 5.2% for 5xFAD/AAV-tau mice; contralateral side: 22.7 ± 6.5% for WT/AAV-tau mice and 24.2 ± 4.6% for 5xFAD/AAV-tau mice).

To further assess the effects of the Aβ pathology, we quantified the distribution of hyperphosphorylated tau (AT8 staining) throughout the hippocampal formation. Ipsilateral to AAV-tau injection, AT8 phosphorylated tau was distributed in the neurites and the soma of neurons (Fig. 1e, f). In the contralateral hippocampus, AT8-positive structures appeared as *puncta* located around Aβ plaques (Fig. 1e, g). As these puncta did not necessarily colocalize with HT7-positive structures, they are likely to also contain phosphorylated forms of endogenous mouse tau. When we compared 5xFAD/AAV-tau and WT/AAV-tau mice, there was a trend towards an increase in the AT8-immunoreactive area in the ipsilateral hippocampus (*p* = 0.088) (Fig. 1h). However, the effects of the Aβ pathology were most prominent in the contralateral side of the hippocampus, where we observed a 5-fold increase of AT8 immunoreactivity (Fig. 1i).

### Overexpression of human WT tau leads to degeneration of the dentate gyrus, an effect which is not enhanced by Aβ pathology

**Fig. 2:**
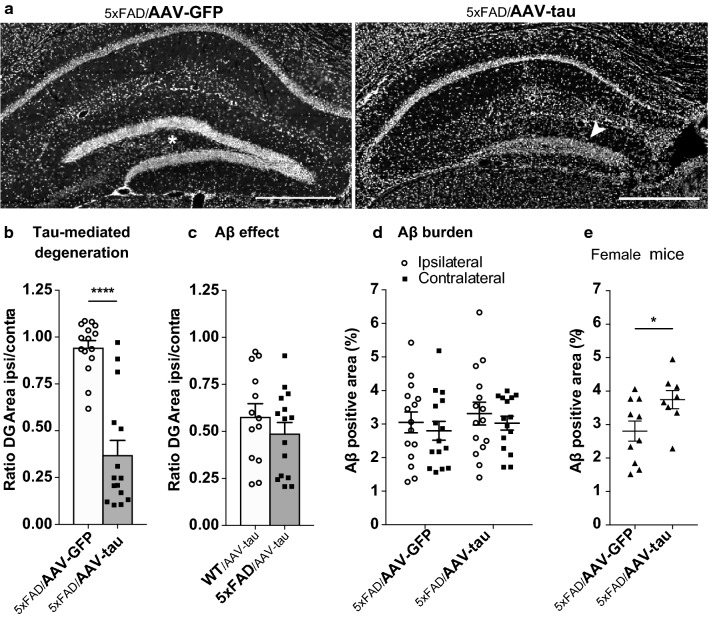
Overexpression of human tau leads to degeneration in the dentate gyrus and increases Aβ deposition in female 5xFAD mice. **a** Representative images of the ipsilateral hippocampus of 5xFAD mice injected with either AAV-tau or the AAV-GFP control vector. Note the degeneration of the dentate gyrus (DG) polymorphic layer and the thinning of the granular layer (white arrowhead) in the hippocampus injected with AAV-tau, in contrast to the hippocampus injected with AAV-GFP (*). DAPI staining in grey. Scale bar: 500 µm. **b** Measured ipsi/contralateral ratio of the DG area showing the effects of AAV-tau on DG degeneration in 5xFAD mice. Note the significant degeneration of the DG induced by AAV-tau injection. **c** Ipsi/contralateral ratio of the DG area comparing WT and 5xFAD littermate mice injected with AAV-tau. **d** Percentage of the ipsi- and contralateral hippocampus covered by Aβ deposits (4G8 staining) in 5xFAD/AAV-GFP and 5xFAD/AAV-tau mice. All mice are included in the analysis. **e** Percentage of the total hippocampus area covered by Aβ deposits (4G8 staining) comparing female mice only. Note the significant effect of AAV-tau on Aβ deposition in female mice. Statistical analysis: two-way ANOVA (d) and unpaired two-tailed Student’s t-test (b, c and e), *p < 0.05 and ****p < 0.0001; 5xFAD/AAV-GFP: n = 15 mice including 10 females, 5xFAD/AAV-tau: n = 15 mice, including 8 females, WT/AAV-tau: n = 13 mice

Next, we sought to further assess the contribution of the tau pathology in this combined Aβ/tau model of AD. Two-months old 5xFAD mice were injected into the CA3 hippocampal region with the AAV-tau vector (5xFAD/AAV-tau) and compared to 5xFAD littermates injected with a similar GFP-expressing AAV8 vector as control (5xFAD/AAV-GFP). Mice were analyzed at 3 months post-injection. In the hemisphere injected with AAV-tau, we noticed a significant hippocampal atrophy, which was most evident in the DG, and was not observed in the contralateral hippocampus. The area normally covered by the polymorphic layer and the thickness of the granular layer were dramatically reduced (Fig. 2a). The induced neurodegeneration was assessed by determining the DG area in the injected hippocampus, as compared to the contralateral non-injected hippocampus. When we compared the 5xFAD/AAV-tau and 5xFAD/AAV-GFP mice, the loss of hippocampal area was observed only in the AAV-tau injected mice and was significantly different from the AAV-GFP group, which indicates that hippocampal degeneration is mainly caused by tau overexpression (Fig. 2b).

To determine if the Aβ pathology further enhanced tau-induced neurodegeneration, we compared the ipsilateral DG area between cohorts of 5xFAD/AAV-tau and WT/AAV-tau mice (Fig. 2c). There was no difference between these groups, suggesting that the Aβ pathology did not affect tau-induced hippocampal neurodegeneration in this model.

### Human tau overexpression does not significantly affect Aβ pathology

In 5xFAD mice, it has been reported in several studies that human mutant tau overexpression can decrease Aβ pathology [10, 24]. However, the effects of human WT tau, which has a lower propensity for pathological aggregation, have been scarcely explored [56]. To determine whether the overexpression of human WT tau could affect Aβ deposition, we compared the hippocampal area covered by amyloid plaques (4G8 signal) in 5xFAD/AAV-tau and 5xFAD/AAV-GFP mice. Overall, there were no differences in Aβ burden between groups, both in the ipsi- or contralateral hippocampus (Fig. 2d). In addition, overexpression of WT human tau did not affect neither the size nor the number of Aβ plaques (Additional file 3: Fig. S3a, b). However, when the analysis was restricted to female 5xFAD mice, we found a significant difference in Aβ burden between 5xFAD/AAV-tau and 5xFAD/AAV-GFP groups (Fig. 2e). In females, but not in males, the Aβ-positive area was significantly increased in the hippocampus of 5xFAD/AAV-tau mice. The same effect was found when measuring the number of Aβ deposits (Additional file 3: Fig. S3b), highlighting sex-dependent differences in the interaction between Aβ and tau pathologies.

### Aβ pathology increases the coverage of the hippocampus by microglial markers and microglia clustering around plaques

**Fig. 3:**
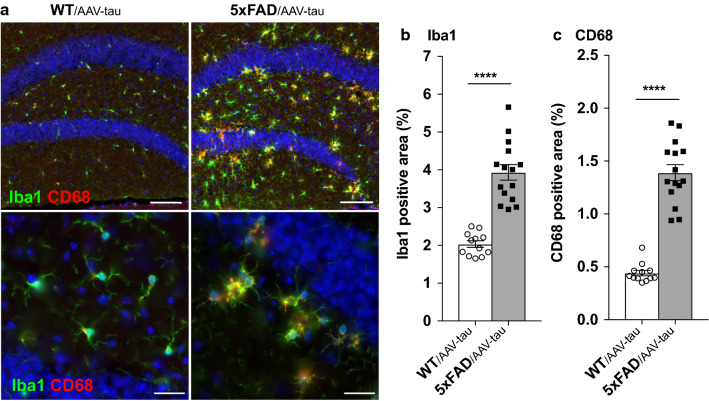
Aβ pathology increases microglia clustering around Aβ plaques. **a** Representative images of Iba1 (in green) and CD68 (in red) staining of microglia in the ipsilateral dentate gyrus in WT/AAV-tau and 5xFAD/AAV-tau mice. DAPI staining is shown in blue. Scale bars: 100 µm (upper panels); 25 µm (lower panels). **b**, **c** Percentage of the hippocampal area covered by Iba1 (**b**) and CD68 (**c**) staining. Note the significant effect of the Aβ on the density of microglial cells. Statistical analysis: unpaired two-tailed Student’s t-test, ****p < 0.0001; WT/AAV-tau: n = 12 mice, 5xFAD/AAV-tau: n = 15 mice

Next, we sought to determine the effects of Aβ and tau accumulation on microglial cells and characterize microglia distribution at neuritic plaques. We used both Iba1 and CD68 markers by immunohistochemistry in the hippocampus to assess the presence of microglia, (Fig. 3a). Iba1 (ionized calcium binding adaptator molecule 1) is a calcium-binding protein broadly expressed by microglia [1, 26], and which is involved in membrane ruffling when microglia pass from a ramified quiescent form to a phagocytic amoebic form [59]. CD68 is a glycoprotein from the lysosomal associated membrane protein (LAMP) which is used as a marker of the macrophage lineage and microglia [25]. We compared 5xFAD/AAV-tau and WT/AAV-tau littermates to assess the effects of the Aβ pathology on the distribution of microglial cells. As expected, the fraction of the hippocampal tissue covered by Iba1 immunoreactivity was significantly increased by 1.95-fold in 5xFAD/AAV-tau mice (Fig. 3b). Furthermore, the abundance of the endosomal/lysosomal CD68 marker was increased by nearly 3.2-fold (Fig. 3c). Both parameters were highly correlated with Aβ burden in the brain tissue, as determined by co-staining with the 4G8 antibody (Additional file 4: Fig. S4a). In addition, analysis of the size and distribution of Iba1-positive cell clusters showed that microglial cells accumulate and form clusters in presence of Aβ plaques in this mouse model of AD (Additional file 4: Fig. S4b, c).

### Human tau overexpression reduces the number of microglia located near Aβ plaques

**Fig. 4:**
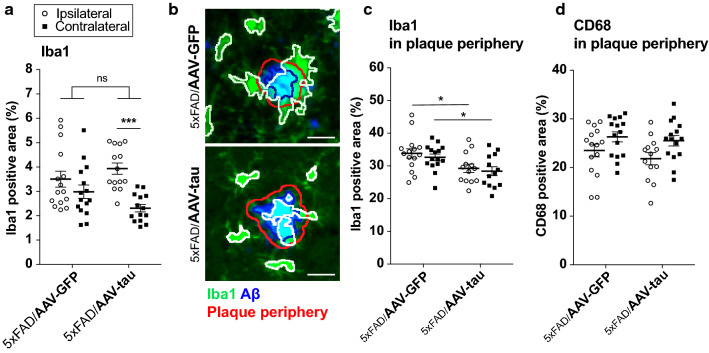
Human tau overexpression affects the clustering of microglia around Aβ plaques. **a** Percentage of the area of the ipsi- and contralateral hippocampus covered by Iba1 positive staining. **b** Representative images of the distribution of Iba1 staining (green) around Aβ plaques (blue) in 5xFAD/AAV-GFP and 5xFAD/AAV-tau mice. To quantify the distribution of microglia in plaque periphery, the region of interest (ROI, delineated by the red line) was defined by including a 5-µm wide rim around the detected Aβ plaque area (periphery shown in blue). The percentage coverage by microglial markers (Iba1 green staining delineated by the white line) was measured within the ROI. **c** Percentage of the ROI area covered by Iba1 staining in both the ipsi- and contralateral hippocampus. Note the significant decrease in the 5xFAD/AAV-tau mice. **d** Percentage of the same ROI area covered by CD68 staining. Statistical analysis: two-way ANOVA with post hoc Sidak’s multiple comparison test; ns non-significant, *p < 0.05, ***p < 0.001; 5xFAD/AAV-GFP: n = 15 mice, 5xFAD/AAV-tau: n = 15 mice

In AD patients and mouse models, aging as well as the accumulation of toxic phospho-tau protein can lead to microglia senescence, possibly contributing to neurodegeneration [9, 29, 68]. We analyzed the effects of WT tau overexpression on the distribution of microglia in the 5xFAD/AAV-tau mouse model. As a control, we used 5xFAD mice injected with AAV-GFP, which showed a very similar accumulation of Iba1-positive microglia in the ipsilateral and contralateral hippocampus (Fig. 4a). In contrast, in the 5xFAD mice injected with AAV-tau, the area covered by the Iba1 staining was significantly different when comparing hippocampal formations in the ipsi- and contralateral hemispheres (Fig. 4a). In the 5xFAD/AAV-tau mice, the accumulation of Iba1-positive cells was most prominent in the injected hippocampus, which is consistent with the neurodegeneration observed in the DG. In the contralateral hippocampus, where tau is mostly present in the axonal projections, the area covered by Iba1-positive microglial cells appeared to be significantly lower than in the ipsilateral hippocampus (Fig. 4a), despite the fact that the Aβ burden was similar in both hemispheres (see Fig. 2d).

Next, we further analyzed the distribution of microglial cells near Aβ plaques. We defined a region of interest corresponding to the Aβ plaque area plus a 5-µm rim (Fig. 4b) and determined in this region the percentage coverage with the microglial markers Iba1 and CD68 (Fig. 4c, d). Remarkably, there was a significant decrease in the plaque coverage by Iba1-positive microglia in 5xFAD/AAV-tau mice as compared to the 5xFAD/AAV-GFP group (Fig. 4c). This effect was apparent both in the ipsilateral and contralateral hippocampus. Tau overexpression was found to relocalize Iba1-positive cells away from Aβ deposits, reducing the interaction between microglia and Aβ plaques. An effect which could disrupt the ability of microglial cells to act as a protective barrier around plaques and thereby increase Aβ toxicity. In contrast, the area covered by the CD68 marker in close proximity to amyloid plaques (Aβ-positive area plus a 5-µm rim) remained similar in both groups (Fig. 4d). These data confirmed that tau overexpression can affect the distribution of microglial cells, an effect most evident at the level of neuritic plaques.

### Passive anti-Aβ immunization via ECT technology delivers mAb11 antibodies in the plasma and brain of 5xFAD mice

Next, we sought to determine the effects of passive anti-Aβ immunization in this model combining amyloid and tau pathologies. To determine the effects of anti-Aβ immunization, we administered the mAb11 antibody using encapsulated cell technology (ECT), as previously described [37]. Briefly, ECT devices consist of a porous polymer membrane used to encapsulate C2C12 myoblasts genetically engineered to secrete recombinant mAb11 anti-Aβ antibodies. The devices were implanted in the subcutaneous tissue of both 5xFAD and WT mice at the age of 12 weeks, one week after injection of the AAV-tau vector in the CA3 hippocampus. In the control sham-treated group, mice were implanted with a capsule loaded with non-modified myoblasts. With mAb11-secreting cells proliferating inside the implant, antibody concentration progressively increased in the plasma of 5xFAD/AAV-tau mice, reaching on average 58 μg/ml at week 20 after implantation (Additional file 5: Fig. S5a). Based on mAb11 concentrations measured every other week, total exposure in each mouse was estimated to reach 4806 ± 2888 μg (mean ± standard deviation) of mAb11 per ml of plasma over 20 weeks of implantation (Additional file 5: Fig. S5b).

Peripherally delivered antibodies can cross the blood brain barrier and bind Aβ plaques in the brain parenchyma [3, 36]. In order to show target engagement of mAb11, we assessed IgG2a immunoreactivity at the level of Aβ plaques using an isotype-specific antibody. In 5xFAD/AAV-tau mice treated with mAb11-secreting ECT, the IgG2a signal labelled Aβ plaques (Additional file 5: Fig. S5c). In contrast, there was no IgG2a immunoreactivity in the sham-treated 5xFAD/AAV-tau mice (Additional file 5: Fig. S5c).

### Passive immunization against Aβ reduces the number and enhances the size and compaction of amyloid plaques in the hippocampus

**Fig. 5:**
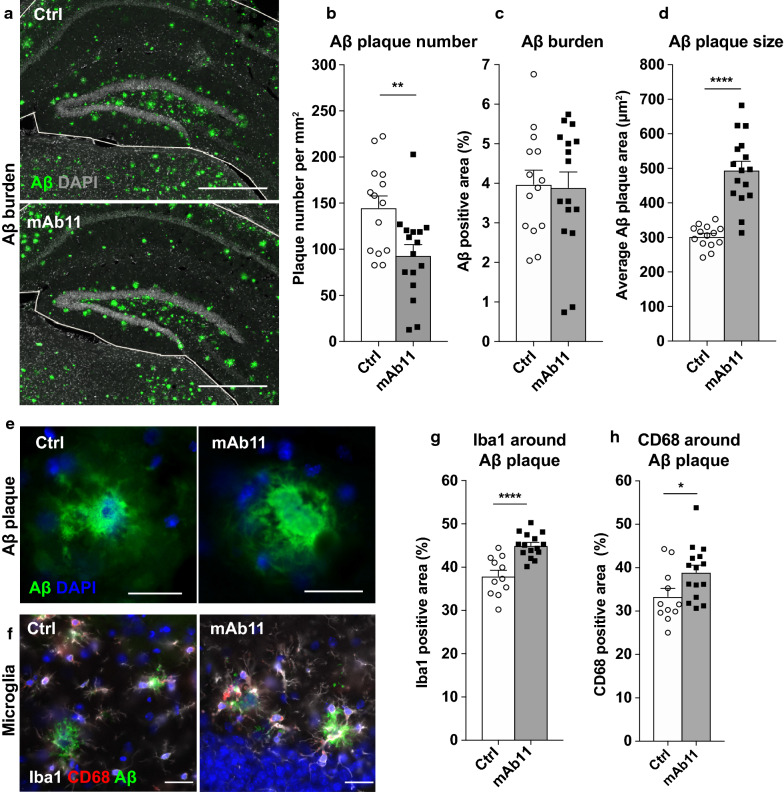
Effects of passive anti-Aβ immunization on Aβ pathology and microglia distribution. **a** Representative images of the Aβ staining (4G8, green) in the contralateral hippocampus of control sham-treated and mAb11-treated 5xFAD/AAV-tau mice. The white line delineates the hippocampal region of interest (ROI) used for analysis of Aβ pathology. DAPI staining is displayed in grey. Scale bars: 500 µm. **b** Number of Aβ positive plaques per mm^2^ detected in the hippocampal ROI. **c** Percentage of the hippocampal ROI covered by Aβ staining. **d** Average area of individual Aβ plaques. Note the significant increase in plaque size in mAb11-treated mice. **e** Representative images of 4G8-positive Aβ plaques in sham- and mAb11-treated mice. Note the lack of the diffuse fibrillary halo in the mAb11-treated condition. DAPI staining is shown in blue. Scale bars: 50 µm. **f** Representative images of Aβ deposits (6e10, in green) surrounded by microglia (Iba1, in white) in a sham-treated and a mAb11-treated 5xFAD/AAV-tau mouse. Shown in red, note the presence of microglia positive for the CD68 phagolysosome marker. DAPI is shown in blue. Scale bars: 25 µm. **g, h** Percentage of the plaque area (including a 5-µm wide rim as described in Fig. 4) covered by the Iba1 staining (**g**) and the CD68 staining (**h**), in the hippocampal region of either control sham-treated or mAb11-treated 5xFAD/AAV-tau mice. Note the significant effects of the mAb11 treatment on Aβ plaque coverage by Iba1- and CD68-positive microglial cells. Statistical analysis: unpaired two-tailed Student’s t-test, *p < 0.05, **p < 0.01, ****p < 0.0001; (**b**–**d**) Control sham-treated 5xFAD/AAV-tau: n = 14 mice, mAb11-treated 5xFAD/AAV-tau: n = 16 mice; (**g**, **h**) Control sham-treated 5xFAD/AAV-tau: n = 11 mice, mAb11-treated 5xFAD/AAV-tau: n = 15 mice

Next, we investigated the effects of passive anti-Aβ immunization on Aβ pathology in the hippocampus of 5xFAD/AAV-tau mice. The delivery of mAb11 by ECT was previously found to decrease Aβ burden in two different Alzheimer’s mouse models [36]. However, this effect was highly dependent on the extent of Aβ pathology at the onset of treatment. As 5xFAD mice develop Aβ plaques already after two months [49], amyloid pathology was already established when the ECT device was implanted at the age of 3 months to deliver anti-Aβ antibodies. Brain tissue was collected 5 months after device implantation and stained using the 4G8 anti-Aβ antibody (Fig. 5a). We measured a significant decrease in the number of 4G8-positive Aβ plaques per mm^2^ in the hippocampus of mAb11-treated 5xFAD/AAV-tau mice compared with the sham-treated group (Fig. 5b). However, the total 4G8-positive area in the hippocampus remained similar in both groups (Fig. 5c). Indeed, the decreased number of Aβ plaques was compensated by a significant increase of plaque size in mAb11-treated 5xFAD/AAV-tau mice, from 303 μm^2^ in the sham-treated mice to an average of 495 μm^2^ in the mAb11-treated mice (Fig. 5d). In addition, Aβ plaques appeared more compact and lacked the fibrillary halo typically observed in non-treated 5xFAD mice (Fig. 5e). Next, we analyzed plaque size distribution (Additional file 6: Fig. S6). Whereas the proportion of large 4G8-positive Aβ plaques (area > 500 μm^2^) nearly doubled in response to mAb11 treatment, the proportion of smaller 4G8-positive Aβ plaques (30 to 500 μm^2^) was significantly decreased. Although the late administration of anti-Aβ antibodies in 5xFAD/AAV-tau mice did not significantly reduce Aβ burden, it was found to modulate Aβ pathology by reducing the number of deposits, while enhancing their size and compaction.

### Passive immunization enhances the coverage of Aβ plaques by microglia in 5xFAD/AAV-tau mice

The anti-Aβ antibody mAb11 was initially designed to bind Aβ plaques and trigger cell-mediated Aβ clearance [4, 78]. In the TAUPS2APP mouse model [21], passive immunization with mAb11 increased the number of Iba1-positive microglia around Aβ plaques [36]. Here, we sought to further explore the effects of passive anti-Aβ immunization on microglia in 5xFAD/AAV-tau mice. We quantified the microglial Iba1 and CD68 markers using immunohistochemistry in the hippocampus of mAb11-treated and sham-treated 5xFAD/AAV-tau mice (Fig. 5f-h). For each animal, the surface of the tissue covered by CD68 or Iba1 immunoreactivity was quantified in the regions of interest, defined as 4G8-immunostained Aβ plaques plus a peripheral 5-µm rim. We measured a significant increase of both the Iba1- and CD68-positive areas around Aβ plaques in mAb11 treated 5xFAD/AAV-tau mice (Fig. 5g, h). These results confirmed that even in presence of overexpressed human tau previously found to reduce the presence of microglia near the amyloid plaques, the anti-Aβ treatment is able to enhance the coverage of Aβ deposits by Iba1 and CD68 positive microglia.

### Passive immunization in 5xFAD/AAV-tau mice reduces the presence of human tau in the ipsilateral hippocampus and prevents its accumulation in dystrophic neurites

**Fig. 6:**
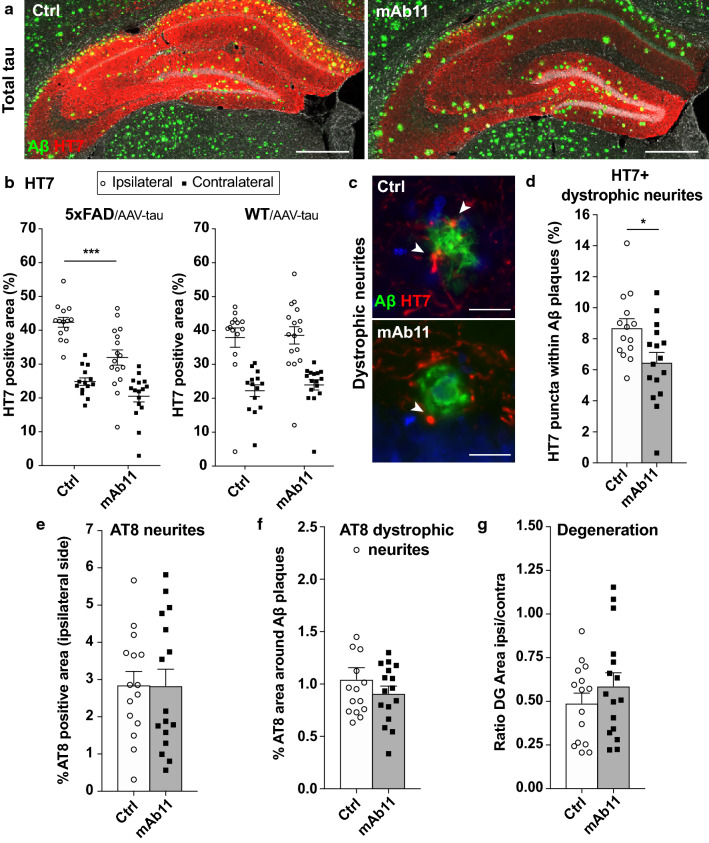
Effect of passive anti-Aβ immunization on human WT tau-induced pathological manifestations. **a** Representative images of human WT tau protein distribution (HT7, in red) in the ipsilateral side of the hippocampus, in a non-treated and a mAb11-treated 5xFAD/AAV-tau mouse. DAPI is shown in grey. Scale bars: 500 µm. **b** Percentage of the ipsi- and contralateral hippocampal region covered by the HT7 staining, showing the effects of the mAb11-treatment in either 5xFAD/AAV-tau or WT/AAV-tau mice. Note the significant decrease of HT7 immunoreactivity in the ipsilateral hippocampus of 5xFAD/AAV-tau mice. **c** Representative images of the HT7-positive dystrophic neurites (red puncta indicated by arrowheads) near Aβ deposits (4G8, in green) in the contralateral hippocampus of a control sham-treated and a mAb11-treated 5xFAD/AAV-tau mouse. DAPI is shown in blue. Scale bars: 25 µm. **d** Percentage of the Aβ-positive area (including a 5-µm wide rim as described in Fig. 4) covered by the HT7 signal, which typically labels dystrophic neurites in the contralateral hippocampus of 5xFAD/AAV-tau mice. Note the significant decrease of the surface covered by human tau positive dystrophic neurites in the mAb11-treated mice. **e** Percentage of the ipsilateral hippocampus covered with AT8 positive signal in control sham-treated 5xFAD/AAV-tau mice compared to mAb11-treated mice. **f** Percentage of the plaque area (5-µm extended Aβ plaque area as described in Fig. 4) covered by AT8 positive puncta in the contralateral hippocampus of sham-treated and mAb11-treated 5xFAD/AAV-tau mice. **g** Hippocampal degeneration assessed by the ipsi/contralateral ratio of the DG area in the sham-treated and mAb11-treated 5xFAD/AAV-tau mice. Statistical analyses: two-way ANOVA with post hoc Sidak’s multiple comparison test (b) and unpaired two-tailed Student’s t-test (**d**–**g**), *p < 0.05, ***p < 0.001; control sham-treated 5xFAD/AAV-tau: n = 14 mice (**b**, **d**) and n = 15 mice (**e**–**g**), mAb11-treated 5xFAD/AAV-tau: n = 16 mice, control sham-treated WT/AAV-tau: n = 14 mice, mAb11-treated WT/AAV-tau: n = 16 mice

Next, we sought to determine the effects of passive anti-Aβ immunization on the hippocampal tau pathology in 5xFAD/AAV-tau mice. Using immunohistochemistry, we quantified the coverage of HT7 human tau immunoreactivity to determine the effects of treatment on tau distribution throughout the hippocampal formation (Fig. 6a). In the ipsilateral side, where the HT7 signal is mostly distributed in the neurites and the soma of neurons, we observed a decrease of the HT7-positive area in the mAb11-treated 5xFAD/AAV-tau mice (Fig. 6b). In contrast, in the hippocampus contralateral to AAV-tau injection where the HT7-positive signal is instead present in the axonal compartment, the percentage of the area covered by human tau was lower and there was no detectable effect of the treatment (Fig. 6b). Importantly, in WT/AAV-tau mice which do not carry any amyloid pathology, the mAb11 treatment had no effects on the extent of the HT7-positive area, neither in the ipsilateral nor in the contralateral hippocampus (Fig. 6b). The latter result shows that the presence of Aβ pathology is required for the mAb11 treatment to induce such an effect. To further assess the consequences of passive anti-Aβ immunization on tau distribution, we quantified the abundance of HT7-positive dystrophic neurites associated to Aβ plaques in the hippocampus contralateral to AAV-tau injection (Fig. 6c, d). Again, we observed a significant reduction of the HT7-positive dystrophic neurites in the mAb11-treated 5xFAD/AAV-tau mice (Fig. 6d), demonstrating a positive effect of the treatment on tau-related pathological manifestations at the level of neuritic plaques.

### Passive immunization does not rescue tau hyperphosphorylation or hippocampal degeneration in 5xFAD/AAV-tau model

Next, we assessed the effects of passive anti-Aβ immunization on the abundance of phosphorylated tau and tau-induced degeneration in the DG. In the hippocampus ipsilateral to AAV-tau injection, AT8 was mostly present in the neurites and neuronal soma of both WT and 5xFAD animals (see Fig. 1e, f). In the contralateral hippocampus however, AT8-positive puncta were found in dystrophic neurites around Aβ plaques, mostly in 5xFAD/AAV-tau mice (see Fig. 1e, g). We compared the area covered by AT8 immunoreactivity in mAb11-treated and sham-treated 5xFAD/AAV-tau mice. There were no difference between these groups, neither in the ipsilateral nor in the contralateral hippocampus (Fig. 6e, f). This despite the fact that HT7 immunoreactivity was previously observed to be reduced (see Fig. 6b). Altogether, these results suggest that late administration of passive anti-Aβ immunization does not affect AT8 immunoreactivity.

In order to determine the effects of anti-Aβ immunization on hippocampal degeneration, we measured the ipsilateral/contralateral ratio of the DG area. The measured ratio was similar in the mAb11-treated and control sham-treated groups of 5xFAD/AAV-tau mice, which suggests that passive anti-Aβ immunization did not alleviate the degeneration of the hippocampus observed in the ipsilateral side (Fig. 6g).

Overall, we found that passive immunization with ECT-mediated delivery of the mAb11 anti-Aβ antibodies effectively clusters microglia around Aβ deposits in 5xFAD/AAV-tau mice. The antibody treatment leads to the compaction of Aβ plaques, prevents accumulation of the human tau protein overexpressed in the hippocampal formation, as well as tau deposition in neuritic plaques.

## Discussion

Here, we assess the effect of passive immunization in a model of AD combining Aβ and tau pathologies in 5xFAD mice. While onset of Aβ plaque deposition is usually observed at the age of 8 weeks in 5xFAD mice, human tau accumulation is induced at week 11 using AAV-tau injection in the CA3 hippocampus. The aim of this model is to induce in Aβ mice the development of the tau pathology, which correlates with cognitive decline in prodromal to mild AD [8]. This is the stage of the disease at which patients have typically been enrolled in clinical trials to assess the effects of passive anti-Aβ immunization [52].

Using this combined mouse model, we assess the respective contributions of Aβ and tau in the pathological changes occurring in the hippocampus. Aβ deposition creates a neurotoxic microenvironment mainly characterized by the formation of neuritic plaques and local recruitment of microglia. On the other hand, tau overexpression triggers clear hippocampal neurodegeneration near the site of AAV-tau vector injection in the dentate gyrus and CA3 hippocampus. Interaction between Aβ and tau pathologies is most evident at the level of the neuritic plaques, with the formation of dystrophic neurites positive for human tau and the presence of phospho-tau positive puncta (AT8). Aβ deposition has recently been shown to promote pathological conversion of tau around plaques, by generating conditions favoring the seeding and spreading of tau aggregates [23, 44]. In those studies, human tau introduced in the mouse brain either by overexpressing a truncated variant of 4-repeat tau [44], or via injection of pathological tau derived from human AD brain [23], was found to form NFTs only in presence of neuritic plaques. Therefore, both tau and Aβ pathologies might be necessary to trigger the pathogenic spreading of human tau in AD.

Overexpression of human tau also affects the deposition of Aβ plaques, an effect which confirms previously reported findings in tau knockout [39] and tau-overexpressing mice [28]. However, changes in Aβ burden are observed only in female mice in our AD model. The reason for this apparent sex-specific response needs to be further explored. However, recent findings may implicate microglia, a cell type with clear sexual differentiation [73]. Indeed, sex modifies the interaction of microglia with Aβ plaques [67], and changes tau-related pathology as a function of the microglial miRNA expression pattern [32].

The presence of Aβ and tau pathologies have seemingly different effects on the distribution and activity of microglial cells. It has been reported that plaques may act as a source of Aβ oligomers and slowly release toxic Aβ species in the brain parenchyma [33]. When recruited to amyloid plaques, protective microglia can form a barrier to reduce toxic Aβ release and locally mitigate pathological manifestations, such as the formation of dystrophic neurites around the plaques [12, 72]. The importance of the barrier function of microglia in sporadic AD is further emphasized by the genetic association with factors such as *TREM2*, which control microglial activity [31, 81]. Indeed, microglia with low TREM2 activity fail to cluster around Aβ plaques, which results in increased neuritic damage [74, 79].

In contrast to Aβ, the effects of tau on microglia activation have been less explored. We find that overexpression of human tau significantly affects the clustering of microglia at the level of Aβ deposits. Overexpression of human tau in the hippocampal formation may therefore relocalize microglial cells away from the sites of Aβ deposition. Consistent with these effects, there is evidence that the chronic presence of pathological tau could induce the premature senescence of microglia, by impairing their function following tau phagocytosis [9, 68]. In addition, neurons with tau aggregates tend to expose the phosphatidylserine phospholipid, an “eat me” signal, causing aberrant phagocytosis [6]. Consequently, the chronic stimulation of microglia exposed to pathological tau species may therefore impair their activity, in particular at the level of the neuritic plaques.

Remarkably, passive anti-Aβ immunization is able to counteract the effects of tau overexpression and increase the coverage of Aβ deposits by microglial cells. Here, the animals were treated by ECT to deliver the mAb11 antibody, which is similar to Gantenerumab and targets oligomeric and fibrillar forms of Aβ to activate cell-mediated removal of the amyloid pathology [4, 36, 78]. This antibody does not recognize soluble monomeric forms of Aβ, which facilitates its target engagement at the level of amyloid plaques in the brain [13]. A recent report also showed that in contrast to Aducanumab, Gantenerumab does not prevent the formation of Aβ42 oligomers [45]. Although passive immunization with mAb11 does not decrease the overall Aβ burden in 5xFAD/AAV-tau mice when administered after the onset of plaque deposition, the treated animals display a lower number of plaques with a larger size. Furthermore, plaques decorated with anti-Aβ antibodies appear more compact, with the loss of the Aβ fibrillary halo. This effect is consistent with previous reports, which showed that passive immunization against Aβ clears mostly small plaques [3] as well as diffuse and fragmented deposits, but not the dense core of Aβ plaques [40, 75–77]. The barrier effect of microglia, with processes tightly surrounding Aβ deposits is likely to contribute to the observed amyloid plaque compaction [12, 79]. Although microglial cells can have detrimental roles by contributing to the production of toxic forms of Aβ and plaque deposition [30, 65, 66], they can also prevent the formation and release of oligomeric Aβ species [12]. AD-associated risk factors are important determinants of microglial activity in response to Aβ plaques [58]. It will be important to determine whether microglial cells clustered at amyloid deposits in response to passive immunization can reduce the release of soluble oligomeric Aβ species from the plaques. By enhancing the barrier effect of microglia and Aβ clearance, passive immunization with antibodies specifically targeting fibrillar forms of Aβ, such as mAb11, might be well suited to prevent the diffusion of toxic soluble Aβ species which affect neuronal function [11, 63].

Among the expected pathogenic consequences of Aβ deposition, the accumulation of pathologic forms of the tau protein at neuritic plaques could play a major role. As an indication that passive immunization can prevent tau-mediated downstream pathogenic effects, we indeed observed a reduction of human tau positive dystrophies. This effect is coherent with a recent study in mice showing that impaired TREM2 activity, which disrupts microglia clustering around Aβ plaques, increases the level of phosphorylated tau in neuritic plaques [43]. This result reinforces the evidence that microglia clustering around Aβ deposits may affect nearby tau pathology. However, when analyzing markers of tau phosphorylation in the mice immunized with mAb11, we found no significant reduction of the AT8 signal. Although other markers of tau hyperphosphorylation need to be analyzed, the treatment might not be sufficient to alleviate all aspects of tau pathology [50]. By limiting tau accumulation at the level of the neuritic plaques, passive anti-Aβ immunization may have broader effects, preventing the conversion of tau towards pathological species, and further spreading and seeding of tau aggregation [44].

Anti-Aβ treatment also reduces the area covered by human tau in the hippocampal formation ipsilateral to the injection of the tau-expressing vector, which is consistent with reduced somatodendritic accumulation of human tau. Mislocalization of tau in the somatodendritic compartment instead of the axon and post-synaptic compartment is thought to be an early manifestation of tau pathology in AD [27]. Tau sorting is a tightly regulated process in neurons, which can be disrupted by neuronal exposure to Aβ oligomers [70, 80]. In primary hippocampal neurons, exposure to Aβ oligomers caused tau missorting through microtubule destabilization [80], disruption of the barrier integrity of the axon initial segment [47, 70], and activation of tau acetylation [70]. Several mechanisms may therefore contribute to the effects of the mAb11 treatment on tau levels in the ipsilateral hippocampus, where tau is mostly located in the somatodendritic compartment of neurons transduced by AAV-tau. Increased microglia activity at the level of neuritic plaques might decrease exposure to Aβ toxic oligomers, in turn reducing tau somatodendritic mislocalization. In addition, anti-Aβ antibodies may reduce intracellular Aβ, which could restore proteasome-mediated degradation and clearance of tau [50, 51, 57, 69]. However, further studies will be needed to unveil the mechanisms involved.

Passive anti-Aβ immunization does not show any significant effects on the tau-induced hippocampal degeneration in our combined Aβ/tau mouse model. It is important to note that neurodegeneration is induced by overexpressing the human tau protein, which may cause toxic effects independent from Aβ deposition. Whereas the two pathologies are likely to be more interdependent in the normal course of AD. However, these results suggest that late administration of anti-Aβ antibodies may not be sufficient to fully rescue the pathogenic effects of tau, suggesting that complementary therapeutic therapies are likely to be needed to further alter the course of AD.

## Conclusion

In an AD mouse model of the Aβ pathology, overexpression of non-mutated human tau in the hippocampal formation leads to tau hyperphosphorylation and accumulation in dystrophic neurites near Aβ plaques. Remarkably, tau affects the clustering of microglia typically observed at neuritic plaques, which may perturb the protective barrier role normally provided by these cells. Continuous administration of the mAb11 antibody binding aggregated Aβ rescues the clustering of microglia at neuritic plaques and enhances plaque compaction. Concomitantly, passive immunization prevents the accumulation of tau in dystrophic neurites and reduces tau levels in the injected hippocampus, where it mainly accumulates in the somatodendritic compartment of long-projection neurons. All together, these results highlight the partial protective effects of anti-Aβ immunization in the complex interaction between Aβ and tau pathologies.

## Supplementary information

**Additional file 1: Figure S1.** Tau and Aβ pathologies in the 5xFAD/AAV-tau mouse model. **a** Representative images of the distribution of Aβ pathology (4G8, in green) and human tau (HT7, in red) throughout the hippocampus of 5xFAD/AAV-tau mice at 1.5 months and 6 months post-vector injection. * indicates the approximate site of AAV-tau injection. Scale bar: 1 mm. **b** Serial sections showing the distribution of Aβ pathology (4G8, in green) and human tau (HT7, in red) throughout the entire hippocampus, 5 months after AAV-tau injection. Coronal sections are shown from the most anterior (top left) to the most posterior (bottom right) regions of the hippocampus. The inter-section distance (200 µm) and the sections selected for immunohistochemical quantification (dashed rectangle) are shown in the most anterior part of the hippocampus. Note the extent of the hippocampal formation covered by human tau over-expression and the overlap of the tau and Aβ pathologies. *Indicates the approximate site of AAV-tau injection. Scale bar: 5 mm. **c** Immunohistochemistry for pathological forms of tau (in red) in the hippocampus of a representative 5xFAD/AAV-tau mouse: misfolded tau (MC1), Ser202/Thr205-phosphorylated tau (AT8), Ser396/Ser404-phosphorylated tau (PHF1). Note the overall somatodendritic localization, as well as differences in the distribution among the various tau species. Scale bar: 100 µm

**Additional file 2: Figure S2.** Amyloid plaques in 5xFAD/AAV-tau mice: markers for microglia and neuritic pathology. Markers of microglia and neuritic pathology at the level of Aβ plaques in the hippocampus of 5xFAD/AAV-tau mice, 5 months after AAV-tau injection. **a** Representative three-dimensional confocal microscopy of an Aβ-positive neuritic plaque (4G8, in green) shows a microglial cell and processes (Iba1, in blue) positive for the CD68 lysosome marker (in red) in close contact with the Aβ deposit. The orthogonal view shows partial colocalization of CD68-positive vesicles and Aβ, indicating phagocytic activity. **b–d** Representative images of neuritic plaques (4G8 or 6e10, in green) surrounded by (**b**) LAMP1 immunoreactivity (red), **c** dystrophic presynaptic neurites (synaptophysin, in red), **d** human tau-positive dystrophic neurites (HT7, in red). **e** Gallyas silver staining (arrowheads) marks neuritic pathology surrounding Aβ plaques (*). Sections are co-stained with DAPI in blue (**b**–**d**) or with nuclear fast red (**e**). Scale bars: 10 µm (**a**) and 25 µm (**b**–**e**)

**Additional file 3: Figure S3.** Tau overexpression in the hippocampus increases the Aβ plaque number in female mice only. **a** Average size of the Aβ deposits (4G8 staining) in the ipsi- and contralateral hippocampus of 5xFAD/AAV-GFP and 5xFAD/AAV-tau mice. **b** Number of Aβ deposits (4G8 staining) per mm^2^ detected in the ipsi- and contralateral hippocampus in 5xFAD/AAV-GFP and 5xFAD/AAV-tau mice. In the right panel, the analysis is restricted to the entire hippocampus (ipsi and contra) of female mice only. Note the significant increase in the number of plaques in the AAV-tau injected group. Statistical analysis: unpaired two-tailed Student’s t-test, **p < 0.01; 5xFAD/AAV-GFP: n = 15 mice, 5xFAD/AAV-tau: n = 15 mice

**Additional file 4: Figure S4.** Aβ burden correlates with microglia activation and triggers microglia clustering. **a** Correlation analysis between the Aβ burden and the Iba1 or CD68 positive area coverage in the hippocampus (% of the hippocampal area covered by positive immunoreactivity). **b** Representative images of a WT/AAV-tau and a 5xFAD/AAV-tau mice illustrating the use of a thresholding algorithm to generate masks of Iba1-positive areas. To measure microglia clustering, contiguous Iba1 positive areas are sorted according to their sizes. In blue: single microglial cells (area < 150 µm^2^); in green: groups of 2 to 3 microglial cells (150 µm^2^ ≤ area ≤ 300 µm^2^); in red: clusters of microglial cells (areas > 300 µm^2^). **c** Microglia cluster size distribution as a percentage of the total number of Iba1-positive areas detected with the antibody. Note the effect of Aβ in 5xFAD mice on the formation of microglia clusters typically located around plaques. Scale bars: 250 µm. Statistical analysis: bivariate Pearson correlation (**a**) and Chi square test (**c**), WT/AAV-tau n = 12 and 5xFAD/AAV-tau n = 15, ****p < 0.0001; WT/AAV-tau: n = 12 mice, 5xFAD/AAV-tau: n = 15 mice

**Additional file 5: Figure S5.** MAb11 anti-Aβ antibody delivery. **a** Average anti-Aβ mAb11 antibody concentration detected in the plasma of 5xFAD/AAV-tau and WT/AAV-tau mice implanted with ECT devices containing C2C12 cells secreting the mAb11 antibody; n = 34 mice. **b** Integrated exposure of the treated mice to the mAb11 anti-Aβ IgG2a antibody over the entire duration of the experiment (144 days). Of note, one animal showed a total mAb11 exposure below the level of detection. As the antibody-secreting myoblasts implanted in this mouse likely failed to survive, this animal was excluded from further analyses. **c** Representative images of mAb11 immunodecoration of Aβ plaques revealed with a specific anti-IgG2a antibody (red staining). In contrast to the positive signal observed at the level of Aβ plaques in the mAb11-treated mouse, there is no detectable presence of IgG2a antibodies in the sham-treated animal. Note that DAPI staining (in blue) labels cell nuclei and cross-reacts with Aβ plaques (*). Scale bars: 25 µm. All mAb11-treated mice included (5xFAD/AAV-tau and WT/AAV-tau): n = 34

**Additional file 6: Figure S6.** mAb11 anti-Aβ antibody affects Aβ plaque size distribution. **a** Plaque size distribution as a percentage of the total number of Aβ-positive areas detected with the 4G8 antibody: yellow for plaques < 30 µm^2^; green for 30-100 µm^2^, pink for 100-500 µm^2^, blue for 500-1000 µm^2^ and red for Aβ deposits > 1000 µm^2^. Areas below 10 µm^2^ were not considered as Aβ plaques. Note the significant increase in the fraction of plaques with a size > 500 µm^2^ following mAb11 treatment. Statistical analysis: Chi square test with control groups as expected distribution, and mAb11 group as observed distribution; control sham-treated 5xFAD/AAV-tau: n = 15 mice, mAb11-treated 5xFAD/AAV-tau: n = 16 mice. **b** Representative images of the masks after thresholding of the Aβ signal for size analysis. Note the presence of large size Aβ deposits with a compact shape (white *) in the mAb11-treated condition. DAPI staining is shown in blue. Scale bars: 100 µm

## Data Availability

The datasets used and/or analyzed during the current study are available from the corresponding author on reasonable request.

## Abbreviations

AD: Alzheimer’s disease
AAV8: serotype 8 adeno-associated viral
Aβ: amyloid beta
CA3: *Cornu Ammonis* areas 3
DG: dentate gyrus
ECT: encapsulated cell technology
IgG: immunoglobulin
mAbs: monoclonal antibodies
NFTs: neurofibrillary tangles
PEG: polyethylene glycol
WT: wild type
5xFAD: 5 human mutations involved in Familial Alzheimer’s disease
