# Supplementary figures and images for: Anti-Aβ antibodies bound to neuritic plaques enhance microglia activity and mitigate tau pathology
Source: Acta Neuropathol Commun. 2020 Nov 23;8:198. doi: 10.1186/s40478-020-01069-3 (PMC7681991; doi:10.1186/s40478-020-01069-3)

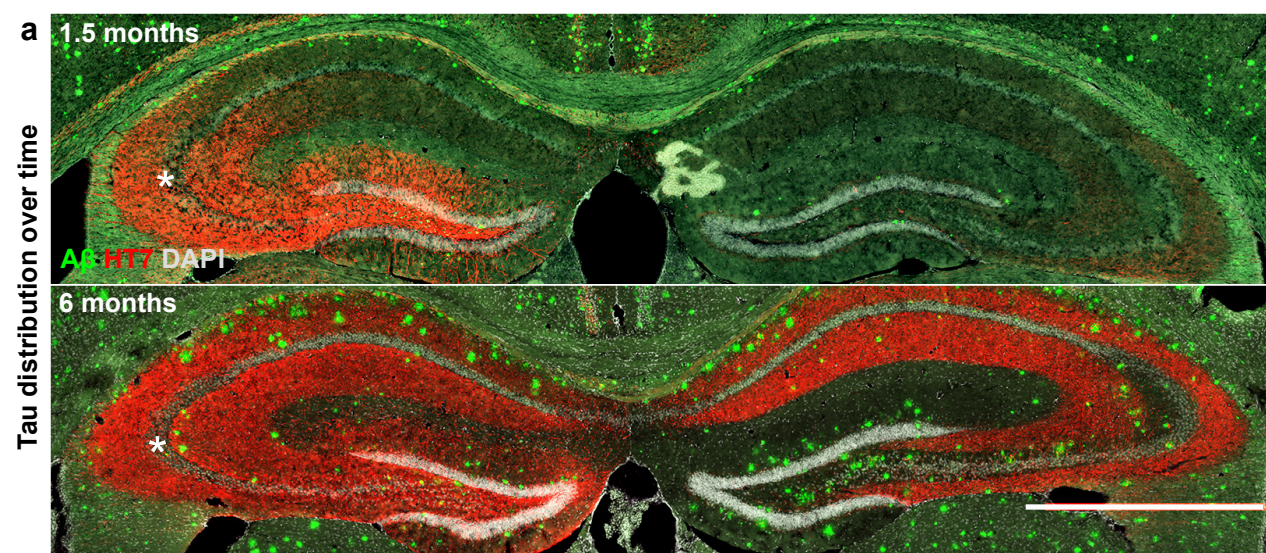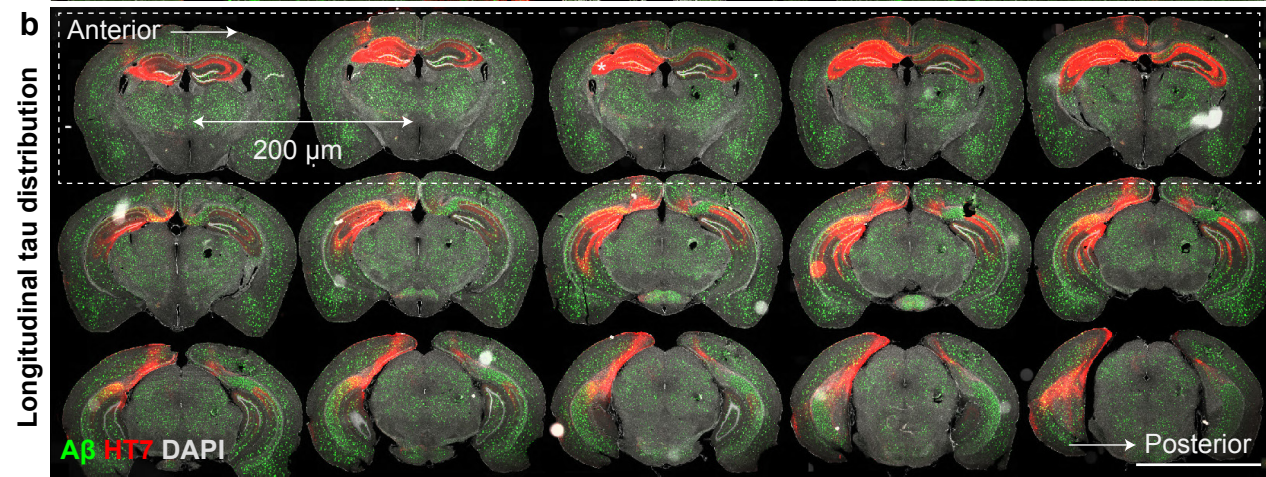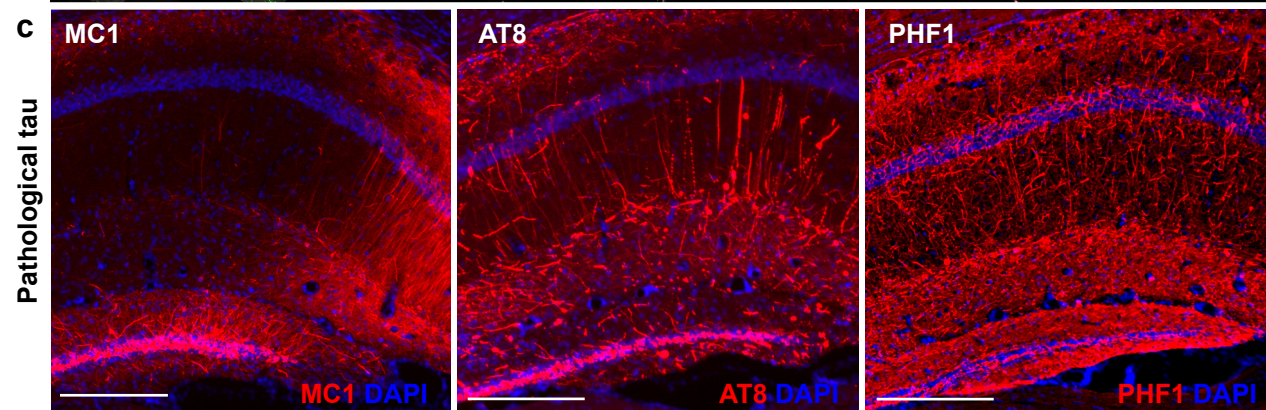

Supplement: Supplementary file 1 — Additional file 1: Figure S1. Tau and Aβ pathologies in the 5xFAD/AAV-tau mouse model. a Representative images of the distribution of Aβ pathology (4G8, in green) and human tau (HT7, in red) throughout the hippocampus of 5xFAD/AAV-tau mice at 1.5 months and 6 months post-vector injection. * indicates the approximate site of AAV-tau injection. Scale bar: 1 mm. b Serial sections showing the distribution of Aβ pathology (4G8, in green) and human tau (HT7, in red) throughout the entire hippocampus, 5 months after AAV-tau injection. Coronal sections are shown from the most anterior (top left) to the most posterior (bottom right) regions of the hippocampus. The inter-section distance (200 µm) and the sections selected for immunohistochemical quantification (dashed rectangle) are shown in the most anterior part of the hippocampus. Note the extent of the hippocampal formation covered by human tau over-expression and the overlap of the tau and Aβ pathologies. *Indicates the approximate site of AAV-tau injection. Scale bar: 5 mm. c Immunohistochemistry for pathological forms of tau (in red) in the hippocampus of a representative 5xFAD/AAV-tau mouse: misfolded tau (MC1), Ser202/Thr205-phosphorylated tau (AT8), Ser396/Ser404-phosphorylated tau (PHF1). Note the overall somatodendritic localization, as well as differences in the distribution among the various tau species. Scale bar: 100 µm [file 40478_2020_1069_MOESM1_ESM.pdf]

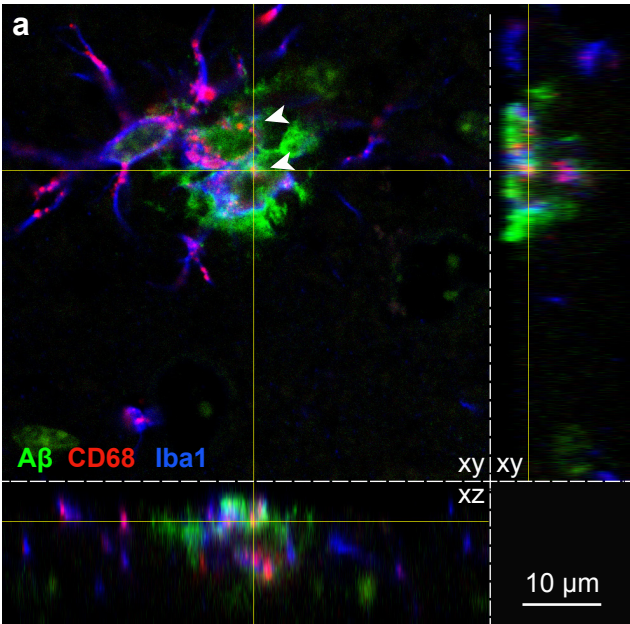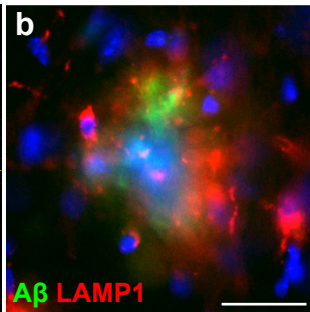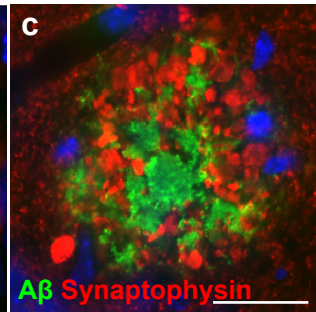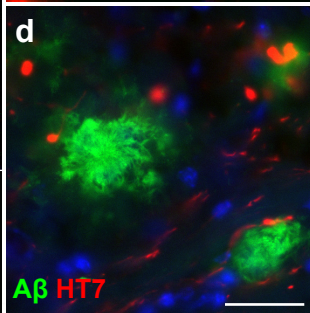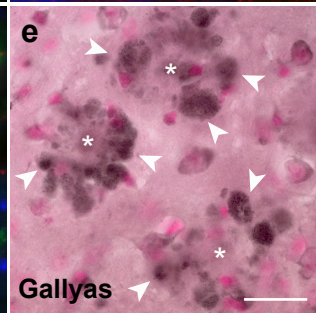

Supplement: Supplementary file 2 — Additional file 2: Figure S2. Amyloid plaques in 5xFAD/AAV-tau mice: markers for microglia and neuritic pathology. Markers of microglia and neuritic pathology at the level of Aβ plaques in the hippocampus of 5xFAD/AAV-tau mice, 5 months after AAV-tau injection. a Representative three-dimensional confocal microscopy of an Aβ-positive neuritic plaque (4G8, in green) shows a microglial cell and processes (Iba1, in blue) positive for the CD68 lysosome marker (in red) in close contact with the Aβ deposit. The orthogonal view shows partial colocalization of CD68-positive vesicles and Aβ, indicating phagocytic activity. b–d Representative images of neuritic plaques (4G8 or 6e10, in green) surrounded by (b) LAMP1 immunoreactivity (red), c dystrophic presynaptic neurites (synaptophysin, in red), d human tau-positive dystrophic neurites (HT7, in red). e Gallyas silver staining (arrowheads) marks neuritic pathology surrounding Aβ plaques (*). Sections are co-stained with DAPI in blue (b–d) or with nuclear fast red (e). Scale bars: 10 µm (a) and 25 µm (b–e) [file 40478_2020_1069_MOESM2_ESM.pdf]

**a A $\beta$  plaque size**

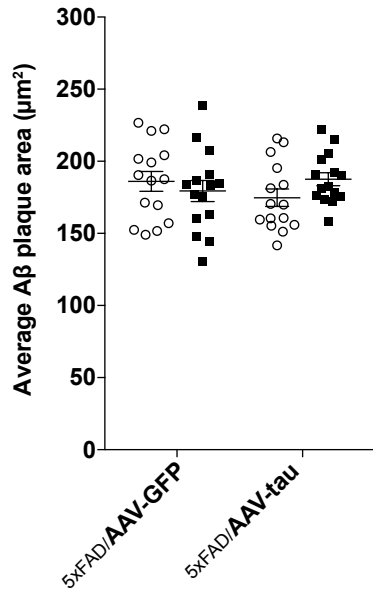

**b A $\beta$  plaque number**

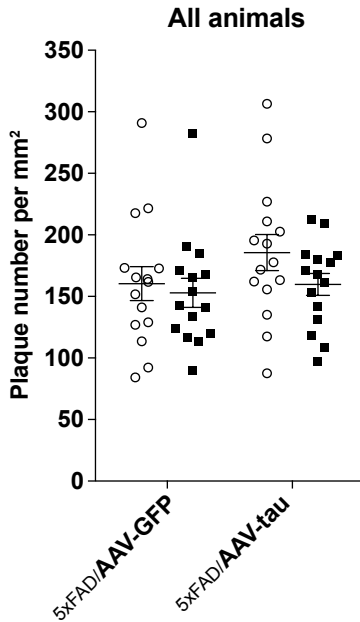

Supplement: Supplementary file 3 — Additional file 3: Figure S3. Tau overexpression in the hippocampus increases the Aβ plaque number in female mice only. a Average size of the Aβ deposits (4G8 staining) in the ipsi- and contralateral hippocampus of 5xFAD/AAV-GFP and 5xFAD/AAV-tau mice. b Number of Aβ deposits (4G8 staining) per mm2 detected in the ipsi- and contralateral hippocampus in 5xFAD/AAV-GFP and 5xFAD/AAV-tau mice. In the right panel, the analysis is restricted to the entire hippocampus (ipsi and contra) of female mice only. Note the significant increase in the number of plaques in the AAV-tau injected group. Statistical analysis: unpaired two-tailed Student’s t-test, **p < 0.01; 5xFAD/AAV-GFP: n = 15 mice, 5xFAD/AAV-tau: n = 15 mice [file 40478_2020_1069_MOESM3_ESM.pdf]

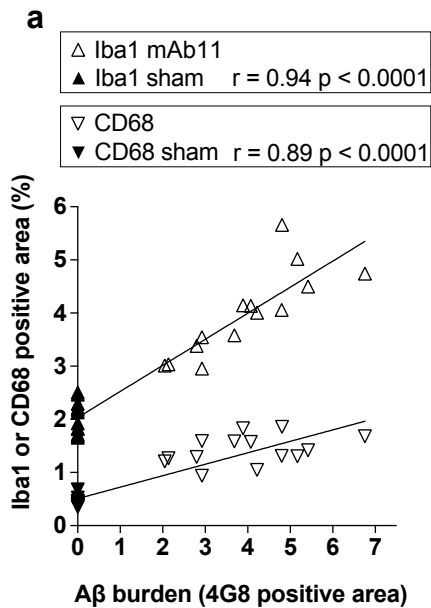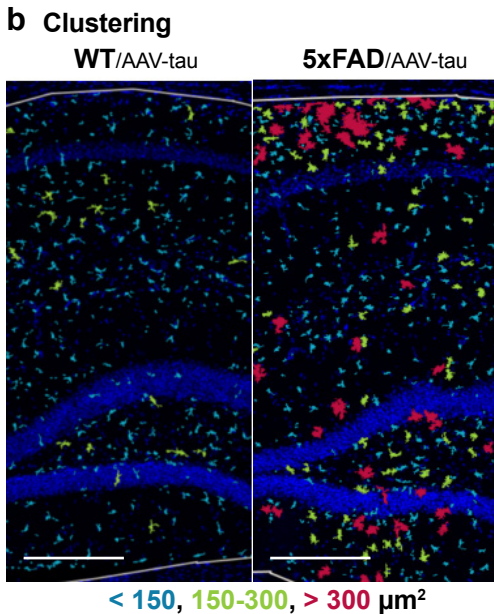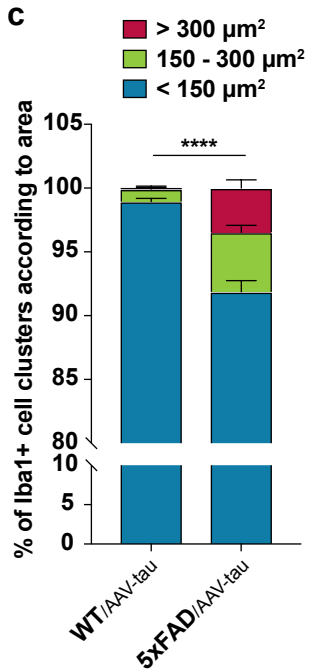

Supplement: Supplementary file 4 — Additional file 4: Figure S4. Aβ burden correlates with microglia activation and triggers microglia clustering. a Correlation analysis between the Aβ burden and the Iba1 or CD68 positive area coverage in the hippocampus (% of the hippocampal area covered by positive immunoreactivity). b Representative images of a WT/AAV-tau and a 5xFAD/AAV-tau mice illustrating the use of a thresholding algorithm to generate masks of Iba1-positive areas. To measure microglia clustering, contiguous Iba1 positive areas are sorted according to their sizes. In blue: single microglial cells (area < 150 µm2); in green: groups of 2 to 3 microglial cells (150 µm2 ≤ area ≤ 300 µm2); in red: clusters of microglial cells (areas > 300 µm2). c Microglia cluster size distribution as a percentage of the total number of Iba1-positive areas detected with the antibody. Note the effect of Aβ in 5xFAD mice on the formation of microglia clusters typically located around plaques. Scale bars: 250 µm. Statistical analysis: bivariate Pearson correlation (a) and Chi square test (c), WT/AAV-tau n = 12 and 5xFAD/AAV-tau n = 15, ****p < 0.0001; WT/AAV-tau: n = 12 mice, 5xFAD/AAV-tau: n = 15 mice [file 40478_2020_1069_MOESM4_ESM.pdf]

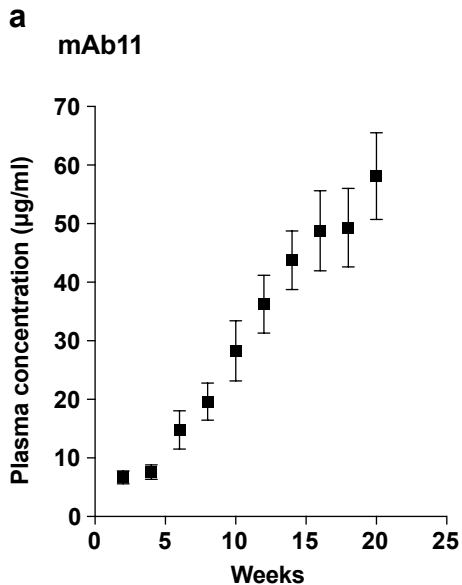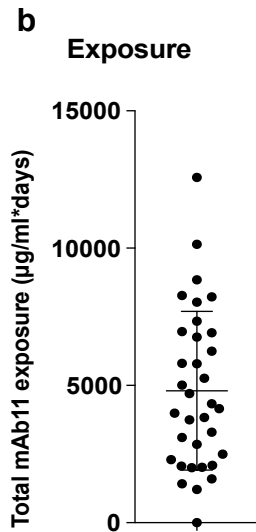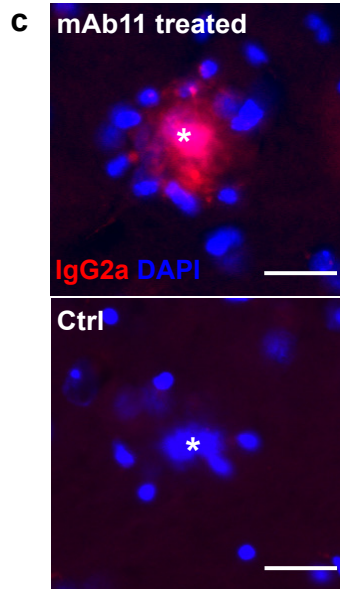

Supplement: Supplementary file 5 — Additional file 5: Figure S5. MAb11 anti-Aβ antibody delivery. a Average anti-Aβ mAb11 antibody concentration detected in the plasma of 5xFAD/AAV-tau and WT/AAV-tau mice implanted with ECT devices containing C2C12 cells secreting the mAb11 antibody; n = 34 mice. b Integrated exposure of the treated mice to the mAb11 anti-Aβ IgG2a antibody over the entire duration of the experiment (144 days). Of note, one animal showed a total mAb11 exposure below the level of detection. As the antibody-secreting myoblasts implanted in this mouse likely failed to survive, this animal was excluded from further analyses. c Representative images of mAb11 immunodecoration of Aβ plaques revealed with a specific anti-IgG2a antibody (red staining). In contrast to the positive signal observed at the level of Aβ plaques in the mAb11-treated mouse, there is no detectable presence of IgG2a antibodies in the sham-treated animal. Note that DAPI staining (in blue) labels cell nuclei and cross-reacts with Aβ plaques (*). Scale bars: 25 µm. All mAb11-treated mice included (5xFAD/AAV-tau and WT/AAV-tau): n = 34 [file 40478_2020_1069_MOESM5_ESM.pdf]

## Size distribution

■ >1000  $\mu\text{m}^2$  ■ 500-1000 ■ 100-500 ■ 30-100 ■ < 30

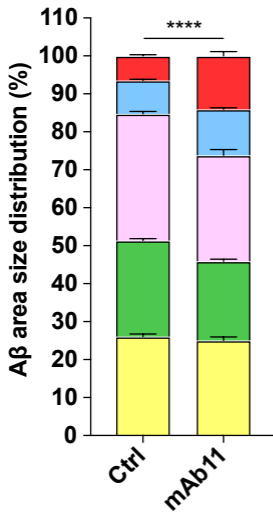

Ctrl

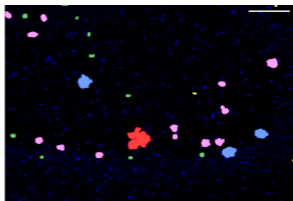

mAb11

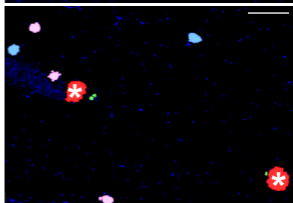

Supplement: Supplementary file 6 — Additional file 6: Figure S6. mAb11 anti-Aβ antibody affects Aβ plaque size distribution. a Plaque size distribution as a percentage of the total number of Aβ-positive areas detected with the 4G8 antibody: yellow for plaques < 30 µm2; green for 30-100 µm2, pink for 100-500 µm2, blue for 500-1000 µm2 and red for Aβ deposits > 1000 µm2. Areas below 10 µm2 were not considered as Aβ plaques. Note the significant increase in the fraction of plaques with a size > 500 µm2 following mAb11 treatment. Statistical analysis: Chi square test with control groups as expected distribution, and mAb11 group as observed distribution; control sham-treated 5xFAD/AAV-tau: n = 15 mice, mAb11-treated 5xFAD/AAV-tau: n = 16 mice. b Representative images of the masks after thresholding of the Aβ signal for size analysis. Note the presence of large size Aβ deposits with a compact shape (white *) in the mAb11-treated condition. DAPI staining is shown in blue. Scale bars: 100 µm [file 40478_2020_1069_MOESM6_ESM.pdf]
